# Prediction of Massive Transfusion in Trauma Patients with Shock Index, Modified Shock Index, and Age Shock Index

**DOI:** 10.3390/ijerph13070683

**Published:** 2016-07-05

**Authors:** Cheng-Shyuan Rau, Shao-Chun Wu, Spencer C. H. Kuo, Kuo Pao-Jen, Hsu Shiun-Yuan, Yi-Chun Chen, Hsiao-Yun Hsieh, Ching-Hua Hsieh, Hang-Tsung Liu

**Affiliations:** 1 Department of Neurosurgery, Kaohsiung Chang Gung Memorial Hospital and Chang Gung University College of Medicine, Kaohsiung City 833, Taiwan; ersh2127@cloud.cgmh.org.tw; 2 Department of Anesthesiology, Kaohsiung Chang Gung Memorial Hospital and Chang Gung University College of Medicine, Kaohsiung City 833, Taiwan; shaochunwu@gmail.com; 3 Department of Plastic Surgery, Kaohsiung Chang Gung Memorial Hospital and Chang Gung University College of Medicine, Kaohsiung City 833, Taiwan; spenc19900603@gmail.com (S.C.H.K.); bow110470@gmail.com (P.-J.K.); 4 Department of Trauma Surgery, Kaohsiung Chang Gung Memorial Hospital and Chang Gung University College of Medicine, Kaohsiung City 833, Taiwan; ah.lucy@hotmail.com (S.-Y.H.); libe320@yahoo.com.tw (Y.-C.C.); sylvia19870714@hotmail.com (H.-Y.H.)

**Keywords:** hypotension, shock, massive transfusion, trauma, injury severity, shock index, modified shock index, age shock index

## Abstract

*Objectives*: The shock index (SI) and its derivations, the modified shock index (MSI) and the age shock index (Age SI), have been used to identify trauma patients with unstable hemodynamic status. The aim of this study was to evaluate their use in predicting the requirement for massive transfusion (MT) in trauma patients upon arrival at the hospital. *Participants*: A patient receiving transfusion of 10 or more units of packed red blood cells or whole blood within 24 h of arrival at the emergency department was defined as having received MT. Detailed data of 2490 patients hospitalized for trauma between 1 January 2009, and 31 December 2014, who had received blood transfusion within 24 h of arrival at the emergency department, were retrieved from the Trauma Registry System of a level I regional trauma center. These included 99 patients who received MT and 2391 patients who did not. Patients with incomplete registration data were excluded from the study. The two-sided Fisher exact test or Pearson chi-square test were used to compare categorical data. The unpaired Student *t*-test was used to analyze normally distributed continuous data, and the Mann-Whitney U-test was used to compare non-normally distributed data. Parameters including systolic blood pressure (SBP), heart rate (HR), hemoglobin level (Hb), base deficit (BD), SI, MSI, and Age SI that could provide cut-off points for predicting the patients’ probability of receiving MT were identified by the development of specific receiver operating characteristic (ROC) curves. High accuracy was defined as an area under the curve (AUC) of more than 0.9, moderate accuracy was defined as an AUC between 0.9 and 0.7, and low accuracy was defined as an AUC less than 0.7. *Results*: In addition to a significantly higher Injury Severity Score (ISS) and worse outcome, the patients requiring MT presented with a significantly higher HR and lower SBP, Hb, and BD, as well as significantly increased SI, MSI, and Age SI. Among these, only four parameters (SBP, BD, SI, and MSI) had a discriminating power of moderate accuracy (AUC > 0.7) as would be expected. A SI of 0.95 and a MSI of 1.15 were identified as the cut-off points for predicting the requirement of MT, with an AUC of 0.760 (sensitivity: 0.563 and specificity: 0.876) and 0.756 (sensitivity: 0.615 and specificity: 0.823), respectively. However, in the groups of patients with comorbidities such as hypertension, diabetes mellitus, or coronary artery disease, the discriminating power of these three indices in predicting the requirement of MT was compromised. *Conclusions*: This study reveals that the SI is moderately accurate in predicting the need for MT. However, this predictive power may be compromised in patients with HTN, DM or CAD. Moreover, the more complex calculations of MSI and Age SI failed to provide better discriminating power than the SI.

## 1. Background

Massive hemorrhage is a major cause of early death in trauma [1]. The rapid and accurate diagnosis of the need for massive transfusion (MT) in bleeding trauma patients is therefore crucial, but remains a challenge [2,3]. Balanced resuscitation with packed red blood cells, fresh frozen plasma, and platelet concentrates has been recommended [1,4,5]. Malone et al. have discussed the various definitions of MT and suggested a MT protocol based on the common definition of transfusion of 10 units of red blood cells in 24 h [6]. In the United States, approximately 85% of major trauma centers have instituted MT protocols to direct the appropriate transfusion of blood products [7]. Many of these scoring systems are complicated and involve results from pathology tests and imaging; therefore, they are often time consuming and not practical for use in the pre-hospital phase [8]. Moreover, some MT protocols are activated by conditions such as existing hemorrhagic shock, large hemoperitoneum, or coagulopathy in trauma patients [1]. Therefore, such MT protocols may not be activated in normotensive patients, those with minimal hemoperitoneum, or those on anticoagulants, despite significant hemorrhage. 

At the time of study, there were 36 models for predicting massive transfusion in trauma, but these are limited by being time-consuming, resource-intensive and scarcely sufficiently validated [9]. Among those models, the most commonly included variable was systolic blood pressure, featuring in all but five models [9]. To identify hypovolemic shock in patients with trauma, in 1967, Allgower and Burri introduced the concept of the shock index (SI), which is the ratio of heart rate (HR) to systolic blood pressure (SBP) [10]. When healthy blood donors were subjected to a defined blood loss of 450 mL, the SI substantially increased whereas the HR and SBP remained within the normal range [11]. The risk for requiring MT in trauma patients rises substantially with elevation of the SI above 0.9 [12]. The risk for requiring MT doubled with a SI > 0.9, quintupled for a SI > 1.1, and was 7 times higher for a SI > 1.3 [12]. The SI is an easily obtained indicator of hemodynamic instability [13,14,15] and a clinical indicator of hypovolemic shock upon arrival at the emergency department (ED), with respect to transfusion requirements and hemostatic resuscitation [16]. Therefore, if a laboratory is not available, assessment of MT requirement based on the SI at the ED for these trauma patients has been suggested [16,17]. A pre-hospital SI ≥ 1.0 after at least 1 L of fluid infusion has a sensitivity and specificity of 47.9% and 90.5%, respectively, for predicting a transfusion of more than five units in 4 h [5]. Vandromme et al. [12] grouped their cohort into six categories based on SI. A SI > 0.9–1.1 had 1.6-fold increased odds (95% CI: 1.13–2.31) for MT. When the SI was greater than 1.1–1.3, the estimated odds for MT increased to 5.57 (95% CI: 3.74–8.30).

Furthermore, the modified shock index (MSI), which is the ratio of HR to mean arterial pressure (MAP) has been proposed to evaluate hemodynamic stability and has served as a better predictor of mortality than the conventional SI [18]. The SBP is replaced by MAP in the equation, so as to include the influence of diastolic blood pressure (DBP) [18]. In a retrospective database review of 22,161 patients who presented to the ED, SI did not correlate significantly with the mortality of emergency patients. However, in patients with normal vital signs at the triage desk, the MSI could be used to identify whether the condition of the patients was critical [18]. The MSI is also an important predictor of mortality and is significantly better than HR, SBP, DBP or SI alone [19]. In addition, because age decreases physiological reserve, age multiplied by the SI—Age SI—substantially increased the discriminating ability of the SI by adjusting for the patient’s age, and served as a better predictor of 48 h mortality compared to HR, SBP, or SI, particularly in old patients [20]. The age-adjusted SI is also able to accurately identify those children who are most severely injured following blunt trauma [21]. In addition, the Age SI had been proposed for predicting the need for a transfusion of ≥4 units in 48 h [20].

A few reports in the literature have evaluated the potential benefits and proposed utilization of the SI as a predictor of need for MT [8,22]. However, no study as yet has focused on evaluating all three indices (SI, MSI, and Age SI) in one population and comparing them with conventional vital sign measurement in trauma patients upon arrival at the hospital. Therefore, the aim of this study was to investigate the potential of the SI and different modified SI derivatives as predictors of the need for MT in trauma patients at the ED of a level I trauma center over a six-year period. 

## 2. Methods

### 2.1. Ethics Statement

This study was pre-approved by the Institutional Review Board (IRB) of the Chang Gung Memorial Hospital (approval number 104-8953C). Informed consent was waived in accordance with IRB regulations.

### 2.2. Study Design

**Figure 1:**
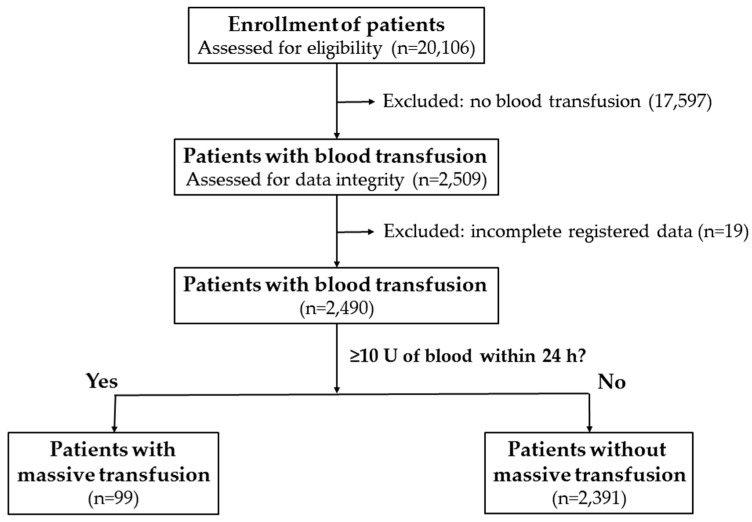
Flow chart of selection of patients with and without massive transfusion.

This retrospective study reviewed data of all 20,106 hospitalized patients registered in the Trauma Registry System of a level I regional trauma center from 1 January 2009, to 31 December 2014 (Figure 1). The study included 2509 patients who had received transfusion of packed red blood cells or whole blood at the ED within 24 h. Patients with incomplete registered data (*n* = 19) were excluded. A patient receiving blood transfusion of 10 units (U) or more within 24 h of arrival at the ED was considered to have received MT (*n* = 99) [8]. The total amount of transfusion included packed red blood cells or whole blood given within the initial 24 h. Other patients who were transfused with less than 10 U of blood within 24 h of arrival at the ED were defined as patients without MT (*n* = 2391). Patients who died on hospital arrival or at the accident scene were not included in the study. Detailed patient information retrieved from the Trauma Registry System of our institution included the following: age; sex; SBP and HR assessed by the nursing staff upon arrival at the triage desk of the ED; SI, defined as the ratio of HR/SBP; MSI, defined as HR divided by MAP = (2 × DBP + SBP) ÷ 3 [18,23]; and Age SI, defined as age multiplied by SI, which accounts for the age of the patient in addition to the factors addressed by SI [20,23]. The pre-existing comorbidities and chronic diseases noted included diabetes mellitus (DM), hypertension (HTN), coronary artery disease (CAD), congestive heart failure (CHF), cerebrovascular accident (CVA), end-stage renal disease (ESRD) and abnormal bodyweight as defined by the World Health Organization [24,25]. Other retrieved data included blood alcohol concentration (BAC); initial laboratory data Hb and BD; ISS; hospital length of stay (LOS); LOS in ICU; and in-hospital mortality. Patients with a BAC of ≥50 mg/dL on arrival at the hospital were considered intoxicated. The ISS was expressed as the median and interquartile range (IQR, Q1–Q3). Odds ratios (ORs) for the associated conditions and injuries of the patients were calculated with 95% confidence intervals (CIs). Adjusted odd ratios (AORs) for mortality adjusted by age, sex, and ISS with 95% CIs were also calculated. The data collected were compared using IBM SPSS Statistics for Windows, version 20.0 (IBM Corp., Armonk, NY, USA). The two-sided Fisher exact test or the Pearson chi-square test was used to compare categorical data. The unpaired Student *t*-test was used to analyze normally distributed continuous data, which was reported as mean ± standard deviation. The Mann-Whitney U-test was used to compare non-normally distributed data. After adjusting for these confounding factors, binary logistic regression was used for evaluating the association of MT with mortality. All possible variables, including SBP, HR, Hb, BD, SI, MSI, and Age SI, were evaluated for cut-off points that could predict the patients’ probability of receiving MT by plotting specific receiver operating characteristic (ROC) curves. The accuracy of each parameter in predicting the investigated outcomes was then calculated in terms of sensitivity and specificity for each possible cut-off. The cut-off point was derived from ROC curves based on the maximal Youden index, which was calculated as sensitivity + specificity − 1, to reflect the maximal correct classification accuracy. The chi-square test was used to verify significant differences between the observed and expected frequencies of investigated outcomes for each possible cut-off value of every parameter. High accuracy was defined as an area under the curve (AUC) of more than 0.9, moderate accuracy as an AUC between 0.9 and 0.7, and low accuracy as an AUC less than 0.7 [26]. *p*-values < 0.05 were considered statistically significant. Linear regression analysis of the level of physiological response and parameters for the units of transfused blood was performed in the patients grouped by sex or the presence or absence of HTN, DM, CAD, or alcohol intoxication. 

## 3. Results

### 3.1. Demographics and Injury Characteristics of Patients Receiving MT

As shown in Table 1, the mean age of patients receiving MT was less than that of those not receiving MT (42.6 ± 18.5 years and 49.4 ± 19.4 years, respectively; *p* = 0.001). Significantly more men than women received MT. Among patients grouped by BMI, no significant differences were found between patients who received or did not receive MT. Incidence rates of HTN were significant lower in the patients receiving MT though no significant difference was seen in incidence rates of other pre-existing comorbidities such as DM, CAD, CHF, and ESRD as compared to patients not receiving MT. There was no significant difference in the incidence of alcohol intoxication (BAC ≥ 50 mg/dL) between patients receiving MT and those not receiving MT.

### 3.2. Injury Severity and Outcome of Patients Receiving MT

A significantly higher ISS was found in patients who received MT than in those who did not receive MT (median (IQR: Q1–Q3), 26 (18–38) vs. 13 (9–20), respectively; *p* < 0.001). When stratified by ISS (<16, 16–24 or ≥25), more patients with an ISS ≥ 25 (30.3% vs. 2.7%, respectively; *p* < 0.001) and fewer patients with an ISS < 16 (15.2% vs. 62.0%, respectively; *p* < 0.001) were found to have received MT. Patients who received MT presented a significantly higher incidence of mortality than those not receiving MT (crude OR 15.8, 95% CI: 9.63–25.94; *p* < 0.001). With confounders including age, sex, and ISS, under control, patients receiving MT presented a significant 5.4-fold increase in mortality rates over those not receiving MT (AOR 5.4, 95% CI: 2.91–9.84; *p* < 0.001). Furthermore, compared to patients not receiving MT, patients who received MT had significantly longer hospital LOS (21.6 days vs. 12.8 days, respectively; *p* < 0.001), a higher proportion being admitted to the ICU (84.8% vs. 36.5%, respectively; *p* < 0.001), and a longer LOS in the ICU (9.7 days vs. 7.3 days, respectively; *p* = 0. 035). 

### 3.3. Association of Physiological Response and Parameters with MT

**Figure 2:**
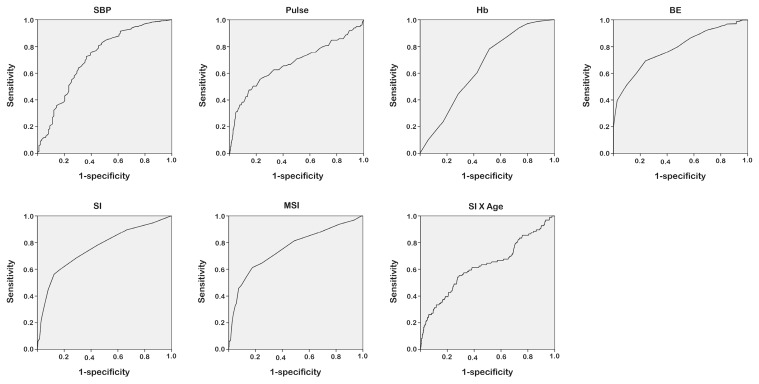
Receiver operating characteristic (ROC) curve analysis to identify different physiological response and parameter cut-off levels for requirement of MT.

Compared to those who did not receive MT, the patients receiving MT presented significantly different changes in physiological response and parameters, including a higher HR and lower SBP, Hb, and BD, as well as increased SI, MSI, and Age SI (Table 2). A logistic regression approach was adopted to evaluate the association between different physiological responses and parameters and the binary outcomes of performing MT. According to the ROC curve analysis, the discriminating powers of the SBP, BD, SI, and MSI were better than would be expected, in the conditions where the AUC was greater than 0.70 (Figure 2). With the sensitivity and specificity of the model for MT set at 0.693 and 0.761, respectively, a BD of −4.50 mmol/L as the cut-off point had the highest AUC of 0.784 (sensitivity: 0.693 and specificity: 0.761). An SBP of 120.5 mmHg, an SI of 0.95, and an MSI of 1.15 was identified as the cut-off for the requirement of MT, with an AUC of 0.716, (sensitivity: 0.725 and specificity: 0.636), 0.760 (sensitivity: 0.563 and specificity: 0.876), and 0.756 (sensitivity: 0.615 and specificity: 0.823), respectively (Table 3). However, the accuracy of prediction of MT was low for all four predictive variables. Further analysis of the cut-off points of SI, MSI, and Age SI was performed in different groups of patients stratified by sex, and absence or presence of HTN, DM, CAD, and alcohol intoxication (Table 4). The results demonstrated that these three indices would still have significant discriminating power in patients of different sex or in the presence or absence of alcohol intoxication. However, in the groups of patients with HTN, DM, or CAD, these three indices had no significant discriminating power in determining the probability of MT. In addition, in those patients who had received MT, none of these variables could be significantly correlated to the amount of blood that was transfused, regardless of stratification according to sex, HTN, DM, CAD, or alcohol intoxication (Supplementary Materials Figure S1).

## 4. Discussion

In this study, the patients receiving MT presented significantly different changes in physiological responses and parameters, including a higher HR and lower SBP, Hb, and BD, as well as increased SI, MSI, and Age SI. However, only four parameters (SBP, BD, SI, and MSI) had a discriminating power of moderate accuracy (AUC > 0.7) as would be expected. The other three parameters, which included HR, Hb, and Age SI, were not acceptable as predictors for MT.

Some authors have observed that even though individual vital signs cannot predict the severity of bleeding, combination of HR and SBP into the SI seems to provide a clinically useful tool for predicting shock in trauma patients [16]. In multivariate logistic regression analysis, the initial SI and HR have been identified as the only variables associated with the requirement for MT, where a much higher odds ratio of SI (OR 9.47; 95% CI: 1.75–51.28; *p* < 0.01) than the odds ratio of HR (OR 1.06; 95% CI: 1.02–1.09; *p* < 0.01) was found [27]. In this study, a BD of −4.50 mmol/L as the cut-off point had the highest AUC of 0.784. This is consistent with previous reports in which BD was an independent predictor of blood transfusion requirement [28,29] and the coagulopathy of trauma appeared to be the combined effect of blood loss, acidosis, hypothermia and clotting factor consumption [30]. In addition, the SI has been reported to provide a clinically useful tool equivalent to the use of BD for predicting shock in trauma patients [16]. In addition, recommendations for the ideal cut-off were varied, with most studies using a cut-off of ≥0.9 to predict hypotension [31] or post-trauma critical bleeding [8]. In this study, the SI, with a cut-off value of 0.95, is moderately accurate in predicting MT. However, because the cut-off value of ≥1.0 (i.e., HR ≥ SBP) was observed to have higher specificity and might be simpler for pre-hospital personnel in particular to calculate [8], the concept of a warning signal (when a trauma patient's SBP is numerically less than his/her HR) makes it easier to identify high-risk patients without requiring any additional equipment [32,33,34].

In this study, the MSI did not present better discriminating power than the SI in predicting MT, and the Age SI was even worse. The result is unsurprising because SBP comprises two-thirds of the value of MAP that is used to calculate MSI and the relationship of diastolic blood pressure to MT is yet to be evaluated. In addition, the Age SI was developed particularly for geriatric patients, with an adjustment for age to increase the discriminating ability of the SI [20]. Zarzaur et al. found that the Age SI performed worse than the SI alone in predicting the need for transfusion of ≥4 units in 48 h, but only when applied to patients ≥55 years old, when the area under the ROC curve was significantly increased from 0.79 to 0.81 [20]. Because no better discriminating power could be provided by the MSI and the Age SI over the traditional SI, the associated unwanted need for complex calculation of these two parameters may limit their use for predicting MT as compared with the traditional SI.

Notably, in this study, although the SI could provide a higher discriminating power to predict MT than SBP or HR, the difference was not great. Moreover, this predictive power may be compromised in patients with HTN, DM, or CAD, in which conditions the dynamic response of HR and SBP and thus the calculated SI may differ from healthy patients and thus hinder its application in predicting MT. To improve the accuracy of the SI, repeated measures at different time points might add more value than a single measure of the SI. A SI averaged over a time period in the pre-hospital setting is better in predicting bleeding [35]. Similarly, an almost five-fold higher mortality (27.6% vs. 5.8%) was associated with an increase of ≥0.3 in observed SI trends from the pre-hospital to the in-hospital setting [14]. 

Unsurprisingly, the use of a single parameter such as the SI to predict the need for MT has limitations in acquiring high accuracy. Various models have been proposed to anticipate MT with variable success [36,37,38,39]. In a validation study of six scoring systems, including Trauma-Associated Severe Hemorrhage (TASH) score [39], Prince of Wales Hospital/Rainer (PWH) score, Vandromme score, Assessment of Blood Consumption/Nunez (ABC) score, Schreiber score and Larsen score, for the risk of MT at a very early stage after trauma [2], the TASH score had the highest overall accuracy as reflected by an AUC of 0.889 followed by the PWH-Score with an AUC of 0.860 [2]. In addition, the Traumatic Bleeding Severity Score (TBSS) has been introduced to accurately predict the need for MT and can be calculated using an iOS application [37]. With a high predictive value (AUC of 0.985, sensitivity 97%, and specificity 96%) [38], the TBSS has strong predictive value and is calculated using only five clinical variables, including the patient’s age, SBP, results of the focused assessment with sonography for trauma scan, the presence/severity of a pelvic fracture, and the serum lactate level [37]. However, this sophisticated calculation can only be done in a hospital with imaging or laboratory facilities, and such models are limited in the urgent prediction of MT in pre-hospital settings or just upon arrival to the hospital. The use of the SI to identify patients at high risk for MT may still provide some valuable information in such conditions, although it has only moderate accuracy and its use in patients with HTN, DM, or CAD may not be appropriate. 

We acknowledge the limitations of our study. First, owing to the retrospective design of this study, there is an inherent selection bias. Second, our trauma registry does not contain information about prior medication such as the use of beta-blockers, antihypertensive agents, analgesics, or drugs for anxiety, which could also influence SBP and HR and therefore the SI. Third, the impact of pre-existing comorbidities on the need for MT remains unclear and could not be excluded from the analysis. Fourth, the amount of blood transfused is grossly based on the clinician’s judgment reflecting current pragmatic practice in trauma resuscitation, rather than being based on an established gold standard for severe hemorrhage. The bias may be greater, particularly when the volume and rate of other fluids infused during resuscitation is unknown. In addition, although restrictive resuscitation or transfusion on the basis of age alone was supported from the literature [40], whether there is a restrictive blood transfusion in the elderly in this study was unknown. Fifth, the analysis of an acute definition of MT (e.g., more than 5 units in 4 h) may be more relevant to the critical condition of the patients, however, such data could not be accurately provided by our Trauma Registry System. Finally, the vital signs and the SI used in this study were based on values recorded at the triage desk; however, due to their dynamic nature, conclusions drawn from a single measurement may differ from those drawn from multiple measurements averaged over time.

## 5. Conclusions

This study reveals that, with a cut-off value of 0.95, the SI is moderately accurate in predicting MT. However, this predictive power may be compromised in patients with HTN, DM, or CAD. Moreover, the more complex calculations of the MSI and the Age SI do not provide better discriminating power than the SI. 

## Figures and Tables

**Table 1:** Demographics and injury characteristics of patients with and without MT.

Variables	MT *n* = 99		No MT *n* = 2391		Odds Ratio (95% CI)		*p*
Age	42.6	±18.5	49.4	±19.4	-		0.001
Sex							
Male	78	(78.8)	1530	(64.0)	2.1	(1.28–3.41)	0.003
Female	21	(21.2)	861	(36.0)	0.5	(0.29–0.78)	0.003
BMI							
BMI < 18.5	5	(5.1)	196	(8.2)	0.6	(0.24–1.48)	0.346
18.5 ≤ BMI < 25	41	(41.4)	1206	(50.4)	0.7	(0.46–1.04)	0.082
25 ≤ BMI < 30	30	(30.3)	636	(26.6)	1.2	(0.77–1.86)	0.418
BMI ≥ 30	9	(9.1)	180	(7.5)	1.2	(0.61–2.48)	0.699
Co-morbidity							
HTN	7	(7.1)	557	(23.3)	0.3	(0.12–0.54)	*p* < 0.001
DM	6	(6.1)	298	(12.5)	0.5	(0.20–1.04)	0.059
CAD	1	(1.0)	58	(2.4)	0.4	(0.06–2.99)	0.516
CHF	0	(0.0)	11	(0.5)	-		1.000
ESRD	0	(0.0)	18	(0.8)	-		0.639
Alcohol ≥ 50	14	(14.1)	226	(9.5)	1.6	(0.88–2.82)	0.161
ISS (median, IQR)	26	(18–38)	13	(9–20)	-		*p* < 0.001
<16	15	(15.2)	1483	(62.0)	0.1	(0.06–0.19)	*p* < 0.001
16–24	26	(26.3)	505	(21.1)	1.3	(0.84–2.10)	0.259
≥25	58	(58.6)	403	(16.9)	7.0	(4.61–10.56)	*p* < 0.001
Mortality (crude)	30	(30.3)	64	(2.7)	15.8	(9.63–25.94)	*p* < 0.001
Mortality (adjusted)	-		-		5.4	(2.91–9.84)	*p* < 0.001
Hospital LOS (days)	21.6	±19.2	12.8	±12.2	-		*p* < 0.001
ICU (*n*, %)	84	(84.8)	872	(36.5)	9.8	(5.60–17.00)	*p* < 0.001
ICU LOS (days)	9.7	±9.8	7.3	±8.2	-		0.035

BMI = body mass index; MT = massive transfusion; CAD = coronary artery disease; CHF = congestive heart failure; CI = confidence interval; DM = diabetes mellitus; ESRD = end-stage renal disease; HTN = hypertension; ICU = intensive care unit; ISS = injury severity score; LOS = length of stay; U = units.

**Table 2:** Physiological response and parameters of patients with and without MT.

Variables	MT		No MT		p
HR (beats/min)	104.8	±29.8	89.3	±20.7	*p* < 0.001
SBP (mmHg)	110.7	±41.4	139.9	±34.8	*p* < 0.001
Hb (g/dL)	11.4	±3.0	13.0	±2.1	*p* < 0.001
BD (mmol/L)	−8.8	±6.3	−3.3	±5.2	*p* < 0.001
SI (bpm/mmHg)	1.1	±0.5	0.7	±0.3	*p* < 0.001
MSI (bpm/mmHg)	1.4	±0.8	0.9	±0.4	*p* < 0.001
Age SI (years × bpm/mmHg)	43.1	±26.3	31.7	±15.2	*p* < 0.001

BD = base deficit; Hb = hemoglobin; HR = heart rate; MSI = mean shock index; SBP = systolic blood pressure; SI = shock index.

**Table 3:** Cut-off value and area under the curve of different physiological variables in predicting the requirement for MT.

Variables	Cut-off	Sensitivity	Specificity	AUC
HR (beats/min)	109.50	0.475	0.853	0.671
SBP (mmHg)	120.5	0.725	0.636	0.716
Hb (g/dl)	11.50	0.782	0.485	0.645
BD (mmol/L)	−4.50	0.693	0.761	0.784
SI (bpm/mmHg)	0.950	0.563	0.876	0.760
MSI (bpm/mmHg)	1.150	0.615	0.823	0.756
Age SI (years × bpm/mmHg)	36.95	0.542	0.723	0.627

AUC = area under curve; BD = base deficit; Hb = hemoglobin; HR = heart rate; MSI = mean shock index; SBP = systolic blood pressure; SI = shock index.

**Table 4:** Cut-off value, sensitivity, and specificity of SI, MSI, and Age SI in predicting MT according to stratification of the groups of patients by the sex, absence or present of HTN, DM, CAD, and alcohol intoxication.

Variables	SI					MSI					Age SI				
	Cut-off	Sensitivity	Specificity	AUC	*p*	Cut-off	Sensitivity	Specificity	AUC	*p*	Cut-Off	Sensitivity	Specificity	AUC	*p*
Male	0.950	0.584	0.862	0.757	*p* < 0.001	1.150	0.636	0.805	0.761	*p* < 0.001	22.550	0.844	0.283	0.626	*p* < 0.001
Female	0.750	0.632	0.745	0.753	*p* < 0.001	1.150	0.526	0.853	0.719	0.001	41.550	0.526	0.830	0.651	0.024
HTN (+)	0.750	0.500	0.874	0.672	0.148	0.750	0.833	0.504	0.653	0.198	40.250	0.667	0.699	0.588	0.458
HTN (−)	0.950	0.589	0.852	0.749	*p* < 0.001	1.150	0.644	0.789	0.748	*p* < 0.001	36.550	0.544	0.749	0.652	*p* < 0.001
DM (+)	0.750	0.667	0.859	0.708	0.081	0.950	0.667	0.785	0.710	0.078	34.350	0.833	0.495	0.636	0.256
DM (−)	0.950	0.578	0.866	0.759	*p* < 0.001	1.150	0.622	0.810	0.756	*p* < 0.001	36.550	0.556	0.732	0.633	*p* < 0.001
CAD (+)	0.950	0.500	0.927	0.661	0.435	0.750	1.000	0.464	0.723	0.282	40.250	1.000	0.609	0.773	0.187
CAD (−)	0.950	0.564	0.873	0.762	*p* < 0.001	1.150	0.617	0.819	0.757	*p* < 0.001	36.950	0.532	0.733	0.628	*p* < 0.001
Alcohol (+)	1.050	0.643	0.815	0.780	*p* < 0.001	1.050	0.929	0.473	0.750	0.002	55.050	0.571	0.869	0.680	0.024
Alcohol (−)	0.950	0.549	0.894	0.753	*p* < 0.001	1.150	0.585	0.847	0.752	*p* < 0.001	36.550	0.524	0.722	0.616	*p* < 0.001

CAD = coronary artery disease; DM = diabetes mellitus; HTN = hypertension; MSI = mean shock index; SI = shock index.

## Supplementary Materials

The following are available online at www.mdpi.com/1660-4601/13/7/683/s1, Figure S1: Correlation of the amount of transfused blood to SI in the patients stratified according to sex, HTN, DM, CAD, or alcohol intoxication.
