# Supplementary material for: Prediction of Massive Transfusion in Trauma Patients with Shock Index, Modified Shock Index, and Age Shock Index
Source: Int J Environ Res Public Health. 2016 Jul 5;13(7):683. doi: 10.3390/ijerph13070683 (PMC4962224; doi:10.3390/ijerph13070683)
Supplement: Supplementary file 1 [file ijerph-13-00683-s001.pdf]

Supplementary Materials: Prediction of Massive Transfusion in Trauma Patients with Shock Index, Modified Shock Index, and Age Shock Index

Cheng-Shyuan Rau, Shao-Chun Wu, Spencer C.H. Kuo, Pao-Jen Kuo, Shiun-Yuan Hsu, Yi-Chun Chen, Hsiao-Yun Hsieh, Ching-Hua Hsieh and Hang-Tsung Liu

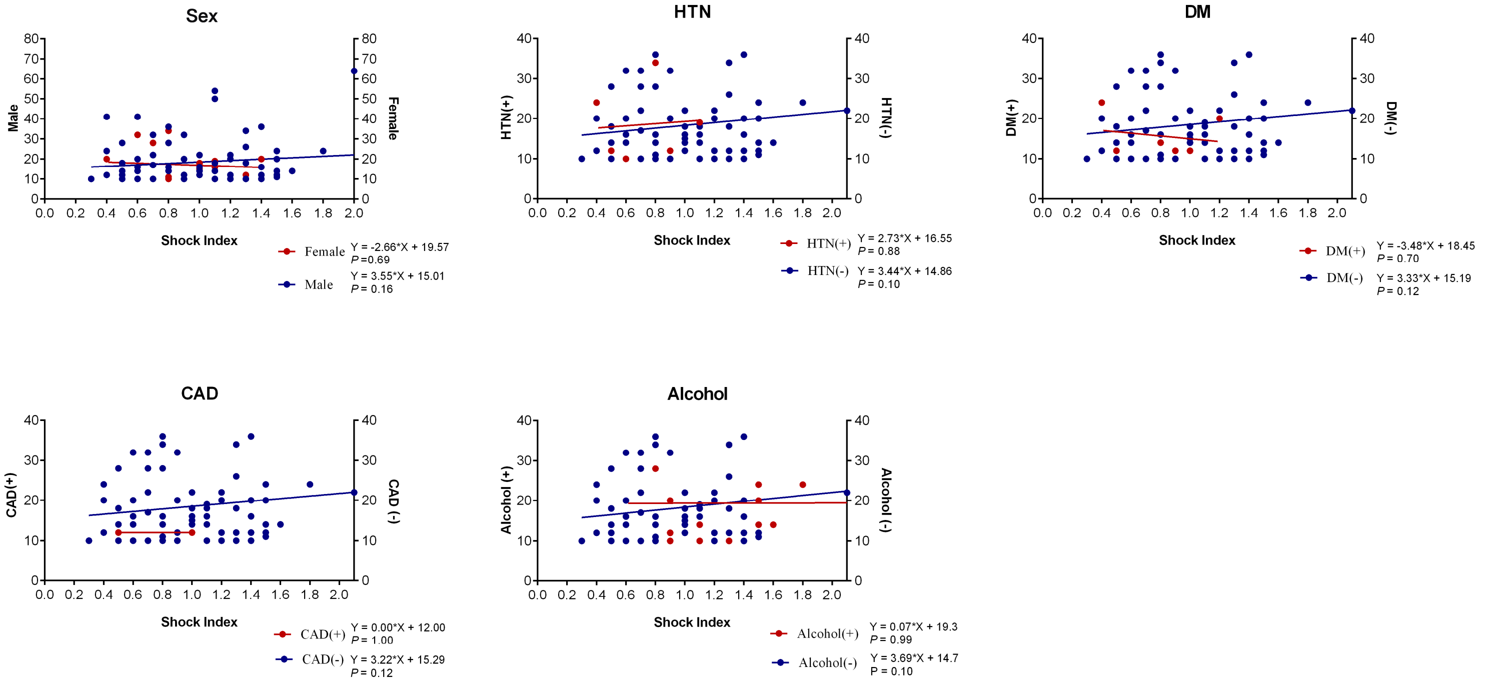

Figure S1. Correlation of the amount of transfused blood to SI in the patients stratified according to sex, HTN, DM, CAD, or alcohol intoxication.

© 2016 by the authors; licensee MDPI, Basel, Switzerland. This article is an open access article distributed under the terms and conditions of the Creative Commons by Attribution (CC-BY) license (http://creativecommons.org/licenses/by/4.0/).
